# Supplementary material for: Extracellular Hsp90 Binds to and Aligns Collagen-1 to Enhance Breast Cancer Cell Invasiveness
Source: Cancers (Basel). 2023 Oct 31;15(21):5237. doi: 10.3390/cancers15215237 (PMC10648158; doi:10.3390/cancers15215237)
Supplement: Supplementary file 1 [file cancers-15-05237-s001.zip › cancers-2611730-supplementary.pdf]

cancers

MDPI

Article

Extracellular Hsp90 binds to and aligns Collagen-1 to enhance breast cancer cell invasiveness.

Supplementary Information

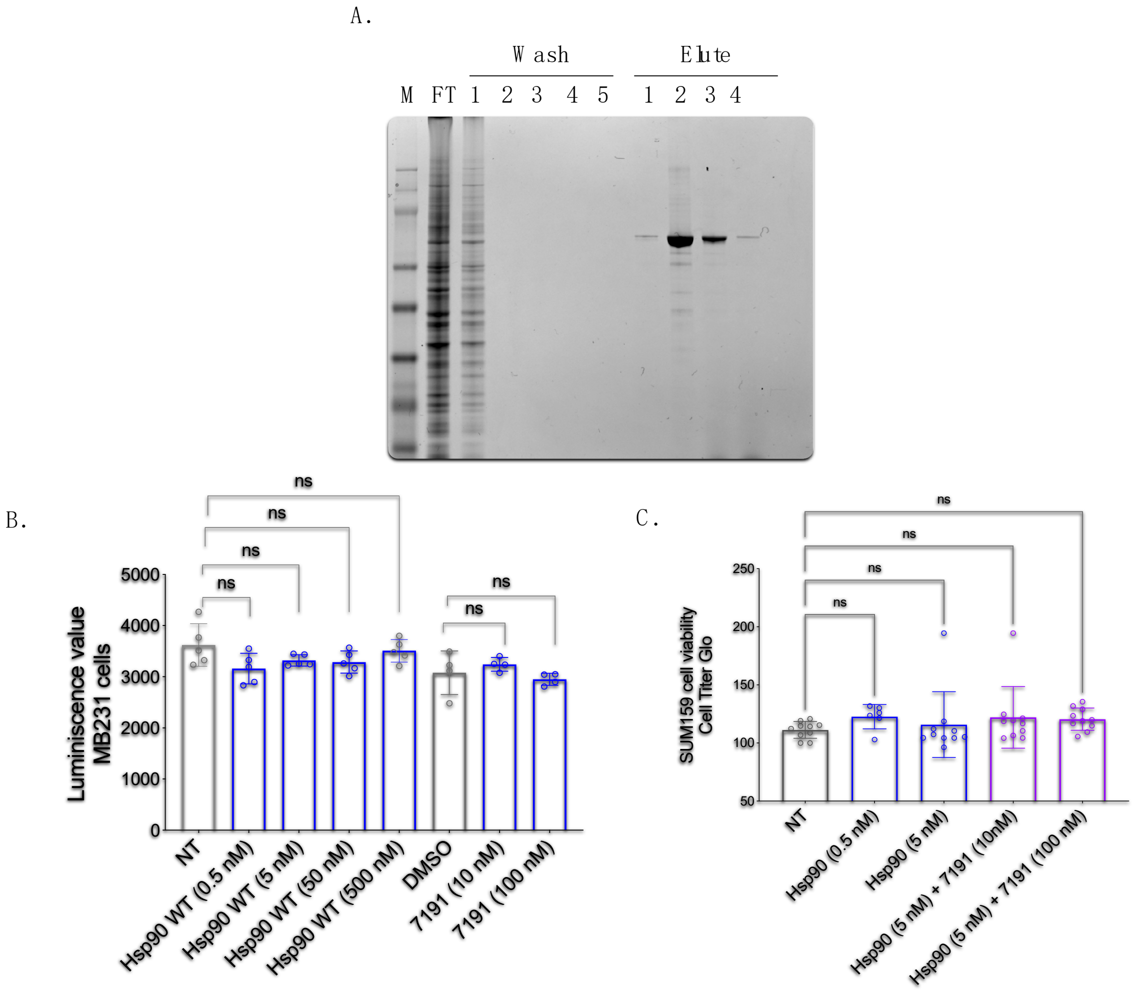

Figure S1. (A.) Coomassie blue staining of FLAG-tag purification samples. M- Novex sharp ladder. FT- flow through after FLAG-M beads were incubated with the cell lysate. **(B.)** and **(C.)** Cell titer glo assay for **(B.)** MB231and **(C.)** SUM159 cells. 10,000 cells and treatment conditions in 100 µl media were added to each 96-well plate and cultured for the duration of the transwell invasion assay (The Cell titer glo experiment was set parallel to the transwell invasion assay). Luminescence was measured after adding equal volume cell titer glo reagent.

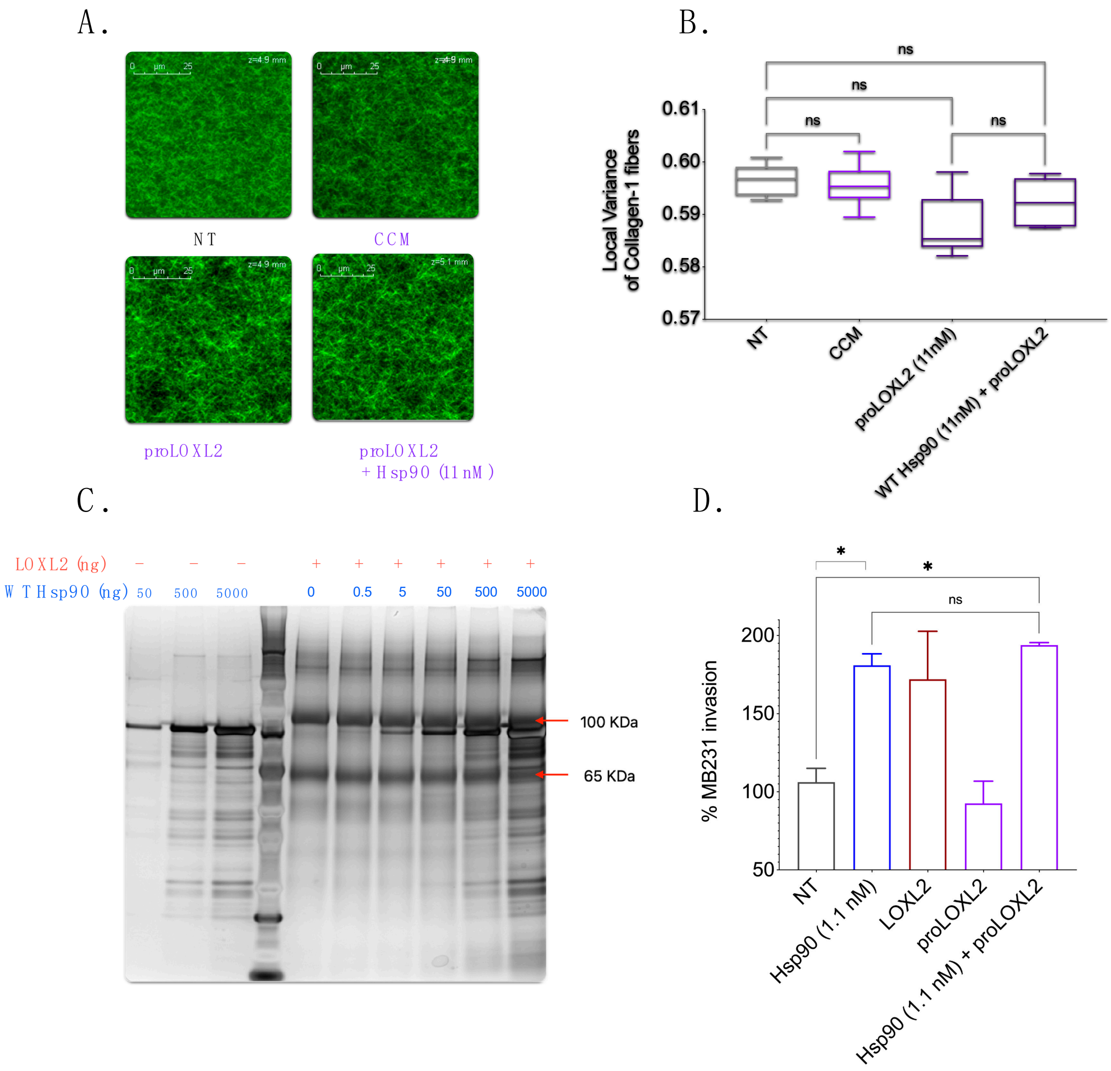

Figure S2. eHsp90 does not increase breast cancer invasion through Collagen-1 by activating LOXL2. **(A.)** The average intensity projection stack of SHG images was obtained SHG imaging experiment with purified proteins treatment of polymerized Collagen gels along with 10X CCM mixed in PBS and incubated for 5 days at 37^0 C. SHG images we acquired, spanning 70 µm vertical distance (1 image/0.5 µm) starting at 200 µm from the base of the gel. The images shown in the figure are representative images of average projections of z-stacks of each condition. **(B.)** Quantification of local variance of the Collagen-1 fiber. (One way ANOVA; P value 0.0148) **(C.)** Silver-stained gel image. Hsp90 and LOXL2 (containing 100 KDa and 65KDa LOXL2; it also had 85 KDa LOXL2) were incubated for 1h, and samples were mixed with reducing loading buffer and run on SDS-PAGE gel. The gels were silver stained and imaged. **(D.)**50,000 MB231 cells were added to the transwell invasion assay's top well with the treatments mentioned. LOXL2 and proLOXL2 were added 11nM/well. The cells were allowed to invade through the Collagen-1 matrix towards 2% FBS media for 8h. (One-way ANOVA; P – 0.0014).

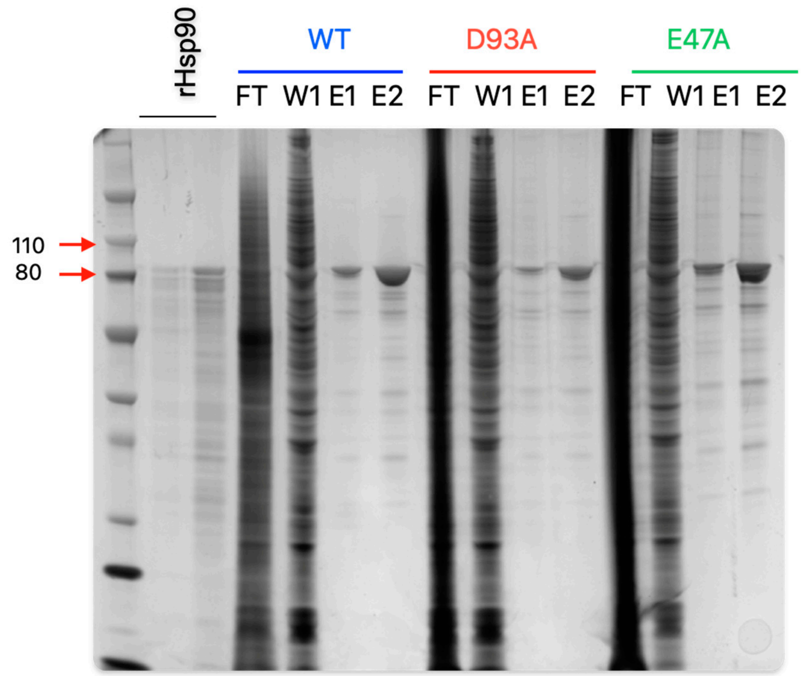

Figure S3. Expression and purification of Hsp90's conformationally restricted mutants in HEK293 cells. **(A.)** The silver-stained gel of purification steps FLAG-tag IP. FT- Flow Through, W1 – Wash 1; E1 and E2 - Elute 1 and Elute 2.

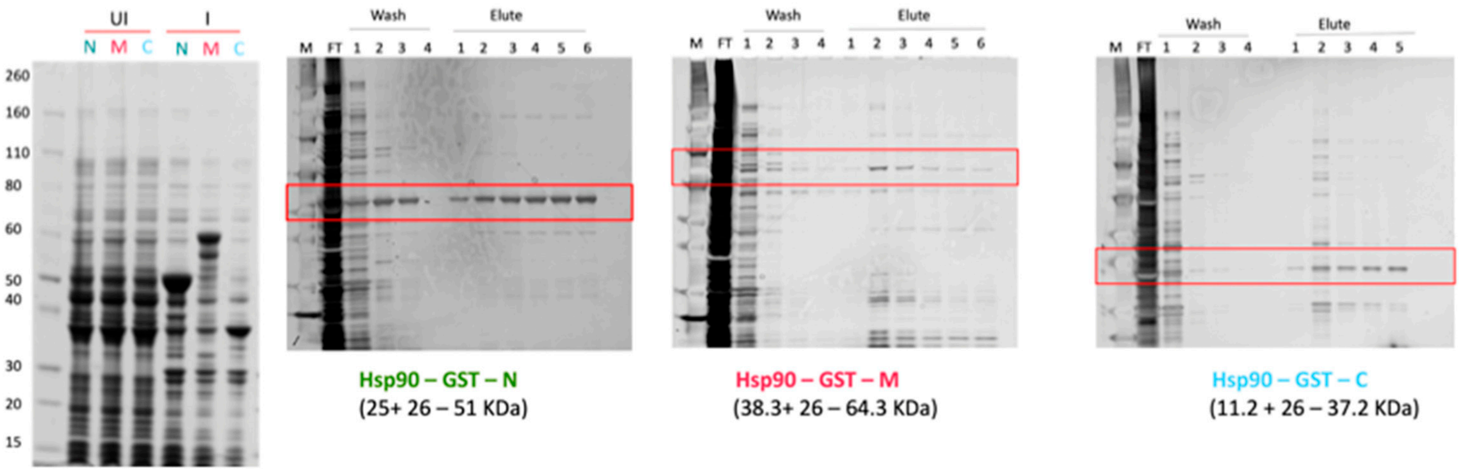

Figure S4. Expression and Purification of domain proteins of Hsp90. **(A.)** The silver-stained gel of lysates obtained from BL21DE3 bacterial cells before and after the induction of domain protein expression. UI- uninduced, I- induced. **(B.)** Silver stain gel of purification steps from GST-tag IP. FT- Flow through.
